# Country ownership and sustainable programming of the HIV response in South Africa: A scoping review

**DOI:** 10.4102/sajhivmed.v24i1.1511

**Published:** 2023-11-30

**Authors:** Refilwe N. Phaswana-Mafuya, Edith Phalane, Haley Sisel, Lifutso Motsieloa, Katherine Journeay, Vuyiseka Dubula, Jabulile Sibeko, Pholokgolo Ramothwala

**Affiliations:** 1 South African Medical Research Council/University of Johannesburg (SAMRC/UJ) Pan African Centre for Epidemics Research (PACER) Extramural Unit, Faculty of Health Sciences, University of Johannesburg, Johannesburg, South Africa; 2 Department of Environmental Health, Faculty of Health Sciences, University of Johannesburg, Johannesburg, South Africa; 3 Key Populations Program, Center for Public Health and Human Rights, Johns Hopkins Bloomberg School of Public Health, Johns Hopkins University, Baltimore, United States of America; 4 South African National AIDS Council, Pretoria, South Africa; 5 Centre for Civil Society, School of Built Environment and Development Studies, University of KwaZulu-Natal, Durban, South Africa

**Keywords:** country ownership, HIV response, sustainable programming, South Africa, political ownership, institutional and community ownership, capabilities, mutual accountability

## Abstract

**Background:**

Concerns have arisen regarding the extent to which South Africa’s HIV response can be country-owned and sustainable given substantial foreign investment and technical support.

**Objectives:**

To assess the extent to which South Africa’s national HIV response is country-owned.

**Method:**

We conducted a scoping review of South African literature using the Global Health Initiative Framework for country ownership.

**Results:**

South Africa has clear aspirations for what should be accomplished and strategies are aligned with national and international priorities. Although South Africa has leveraged community-based strategies to reach key populations (KPs), most are supported by international donors, which poses a sustainability challenge. Despite robust capacity strengthening and training programmes, South Africa continues to face healthcare worker shortages. While it is commendable that South Africa funds nearly 70% of the national HIV response, the funds mainly support HIV treatment. This may create dependency on international partners.

**Conclusion:**

South Africa appears to be progressing well along the spectrum of country ownership, but sustained efforts are required to combat HIV. Greater ownership over KP programming and prevention services are especially needed to achieve greater impact.

**What this study adds:** To the best of the authors’ knowledge, this article provides the first analysis of the country ownership of South Africa’s HIV response. This review provides insights into gaps in country-owned HIV programming and practical recommendations for closing them to improve the national response. The highly consultative and collaborative nature of this scoping review warrants trust in the comprehensiveness of the evidence presented.

## Introduction

Country ownership and sustainable programming of the HIV response came to the forefront of discussions on international investments in health and development in the early 2000s. For example, the 2005 Paris Declaration cites country ownership as one of the core tenets of aid effectiveness.^1^ Similarly, the Accra Agenda for Action highlights the importance of country ownership, stating that international donors will provide support to developing governments to lead and implement their country priorities.^2^ The South African National AIDS Council (SANAC) works with the South African government (SAG), civil society (including key populations [KPs] such as female sex workers [FSWs], transgender populations, men who have sex with men [MSM], people who are incarcerated, and people who use drugs [PWUD]), as well as the private sector, to coordinate the development and implementation of the National Strategic Plan (NSP). The newly launched fifth NSP for HIV, tuberculosis (TB) and sexually transmitted infections (STIs) is in effect from 2023–2028.^3^ The NSP is a critical tool in ending HIV as a public health problem in South Africa, which bears the largest HIV epidemic in the world with nearly eight million people (13.5% of the population) living with HIV (PLHIV) in 2022.^4,5^

In addition to coordinating government programmes, which is spearheaded by SANAC, the United States President’s Emergency Plan for AIDS Relief (PEPFAR) is one of the largest actors in the HIV response, funding 24% of all HIV programmes in South Africa in 2020.^6,7^ The Global Fund to Fight AIDS, Tuberculosis and Malaria has also made a significant contribution.^7,8^ Despite the invaluable contributions of international donors, justifiable concerns have arisen regarding the extent to which South Africa’s HIV response can be country-owned and sustainable given substantial foreign investment and technical support.

Reflecting on the importance of championing a sustainable HIV programme in South Africa, the SAG works in tandem with local and global partners to create a bi-annual Sustainability Index and Dashboard (SID).^9^ The latest SID completed in November 2021 showed that 10 of the 17 critical indicators in the HIV response were considered sustainable. Notable vulnerabilities were identified in several areas: ‘policies and governance, civil society engagement, service delivery, human resources for health, quality management, epidemiological and health data, performance data, and data for decision-making ecosystem’.^9^ However, these topic areas have not been evaluated using the Global Health Initiative’s (GHI) criteria for country ownership.

The GHI produced a conceptual framework that defines and characterises four dimensions of country ownership: (1) political ownership and stewardship; (2) institutional and community ownership; (3) capabilities; and (4) mutual accountability, including finance.^8^ By their definition, country ownership is a spectrum ‘characterised by government, communities, civil society and private sector able to lead, prioritise, implement and be accountable for a country‘s health response’.^8^

This article assesses the extent to which South Africa’s national HIV response is country-owned, according to the GHI framework. This article may strengthen the national HIV response by providing insights into gaps in country-owned HIV programming and practical recommendations for closing them. This is feasible, provided that South Africa considers and implements the findings from this article. A multisectoral response remains critical as no single agency can effectively respond sustainably to the HIV epidemic alone. This review has implications for future pandemic preparedness.

## Methods

We conducted a scoping review of available literature and reports (both published and unpublished) on HIV programmes in South Africa to ensure a comprehensive and up-to-date review. In addition, a manual search on the SANAC, Human Sciences Research Council, The Joint United Nations Programme on HIV/AIDS (UNAIDS), and local implementing partners’ websites was conducted. The following search terms were used: ‘political ownership and stewardship in South Africa’, ‘institutional and community ownership in South Africa’, ‘capabilities’, ‘mutual accountability’, ‘finance’, ‘South Africa’, ‘HIV response in South Africa’, ‘country ownership in South Africa’. However, it should be noted that the search terms did not elicit adequate information as some reports were not available in the public domain. We relied on technical reports, annual reports, policies, notes, and related programmatic information provided by stakeholders, some of whom served as co-authors. Given the fact that this was a scoping rather than a systematic review, we do not claim that all documents relating to the subject were used. However, care was taken to ensure that key documents, as provided by stakeholders, were used.

**FIGURE 1:**
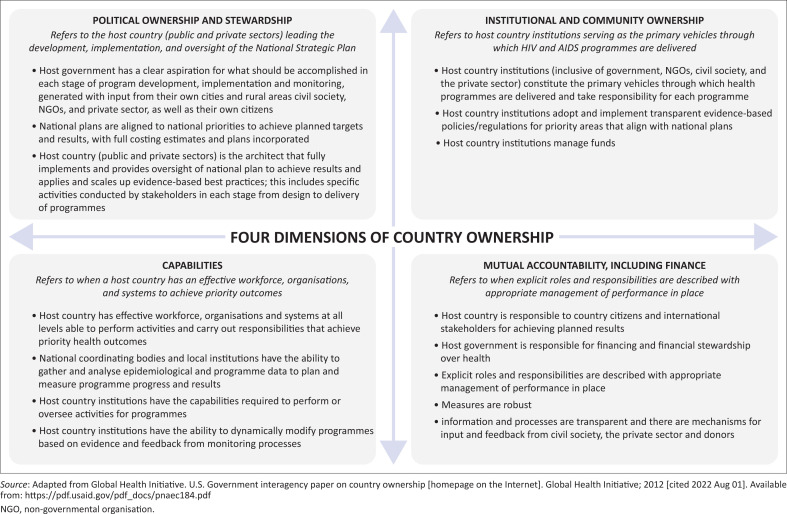
Global Health Initiative’s four dimensions of country ownership.

Data have been presented in tables where applicable. RefWorks software was used to manage the articles and reports that were identified. After identifying relevant documents to include in our analysis, we used thematic content analysis to apply the GHI criteria for meeting the four dimensions of country ownership (Figure 1)^8^ to the available evidence.

### Ethical considerations

This scoping review falls under the umbrella study entitled ‘Harnessing big heterogeneous data to evaluate the potential impact of HIV responses among key populations in generalized epidemic settings in sub-Saharan Africa (The Boloka Project)’, which was approved by the Research Ethics Committee of the Faculty of Health Sciences, University of Johannesburg (REC-1504-2022). This review included the use of published and unpublished literature.

## Results

The evidence of South Africa’s country ownership of the HIV response, according to the Global Health is available in Table 1.

**TABLE 1:** Evidence of South Africa’s country ownership of the HIV response, according to the Global Health Initiative’s 2012 Country Ownership Conceptual Framework.

Criteria	Progress
**Dimension 1: Political ownership and stewardship**	
1.1 Host government has a clear aspiration for what should be accomplished in each stage of programme development, implementation and monitoring, generated with input from their own cities and rural areas, civil society, NGOs, and private sector, as well as their own citizens.	Goals and aspirations are clearly outlined. Signatory to international and regional declarations; explicit policies and commitments for KPs.^4,10^ Local input is welcomed and valued. Consultations (online survey and face-to-face) at the national and provincial levels; open call for submissions and comments; input from various stakeholder groups and technical teams (e.g., Prevention Technical Task Team, Social and Structural Drivers Technical Task Team, Human Rights Technical Task Team, Strategic Information Technical Task Team).^4^
1.2 National plans are aligned to national priorities to achieve planned targets and results, with full costing estimates and plans incorporated.	National plans and priorities are aligned. The NSP has concrete goals, each with corresponding targets and activities; phased approach with mid-term targets to ensure goals are attainable.^4^ Costing estimates are provided. The 2023–2028 NSP has a detailed costing for activities; FINCAP assists provinces in creating more accurate budgets and tracking expenses.^4^
1.3 Host country (public and private sectors) is the architect that fully implements and provides oversight of national plan to achieve results and applies and scales up evidence-based best practices; this includes specific activities conducted by stakeholders in each stage from design to delivery of programmes.	South African organisations are responsible for creating and overseeing the implementation of the NSP. Interventions prioritised by the NSP are evidence-based and are promoted by global health authorities (e.g. WHO, UNAIDS).^3^
**Dimension 2: Institutional and community ownership**	
2.1 Host country institutions (inclusive of government, NGOs, civil society, and the private sector) constitute the primary vehicles through which health programmes are delivered and take responsibility for each programme.	Local institutions (e.g., AFSA, Beyond Zero, NACOSA, Aurum, TB/HIV Care, Right to Care, Ritshidze) are responsible for delivering services; however, they are primarily supported by international donors.
2.2 Host country institutions adopt and implement transparent, evidence-based policies/regulations for priority areas that align with national plans.	Policies are evidence-based and in alignment with national plans. Priority areas include improving ART adherence and retention, reducing HIV-related deaths, and reducing stigma and discrimination faced by PLHIV; community service delivery approaches are championed.
2.3 Host country institutions manage funds.	Local implementing partners often receive donor funding (e.g., PEPFAR, Global Fund) and are responsible for managing these funds. Local institutions may serve as grant managers, determining which sub-recipient organisations will receive funding based on funders and government policies and guidelines; annual budgets and reports are published to promote transparency.
**Dimension 3: Capabilities**	
3.1 Host country has effective workforce, organisations, and systems at all levels able to perform activities and carry out responsibilities that achieve priority health outcomes.	There are substantial workforce challenges that jeopardise South Africa’s ability to address the demand for HIV services. The high demand for HIV services outpaces the available human resources, particularly for KPs who may require differentiated service delivery. However, there are robust capacity strengthening programmes on human rights, non-discrimination, violence prevention, and gender-based violence. Procurement systems are insufficient to ensure timely access to medical supplies. Supply chain issues have manifested in stockouts, for example of PrEP.
3.2 National coordinating bodies and local institutions have the ability to gather and analyse epidemiological and programme data to plan and measure programme progress and results.	South Africa has several data systems (e.g., DHIS, TIER.net, NHLS CDW) that measure programme progress and results. Current data systems tend to be siloed and duplicative; the exclusion of unique identifiers for KPs poses challenges to disaggregating data and understanding progress among different priority groups.
3.3 Host country institutions have the capabilities required to perform or oversee activities for programmes.	SANAC and other South African organisations are well equipped to perform and oversee activities. Several mechanisms exist for overseeing programme activities, including PRC and the M&E team at SANAC, which is responsible for producing the GAM reports.^6,10^
3.4 Host country institutions can dynamically modify programmes based on evidence and feedback from monitoring processes.	Feedback on the progress made toward NSP targets is regularly generated. There is routine monitoring of HIV indicators; SANAC’s new Situation Room will allow for real-time monitoring and decision-making.
**Dimension 4: Mutual accountability, including finance**	
4.1 Host country is responsible to country citizens and international stakeholders for achieving planned results.	The SAG is responsible for carrying out activities and sharing results transparently to both citizens and other stakeholders. SANAC, Operation Phutuma, and the Global Fund Country Coordinating Mechanism are three examples of how the SAG and stakeholders are engaged in ongoing dialogue.^4,10^
4.2 Host government is responsible for financing and financial stewardship over health.	The SAG is the primary funder of the national HIV programme. In FY 2019–2020, the SAG funded 69% of the NSP; the RMC provides NSP spending oversight.^4^
4.3 Explicit roles and responsibilities are described with appropriate management of performance in place.	SANAC encourages multisectoral collaboration, allowing for the optimal allocation of responsibilities. SANAC works closely with donors and multilateral organisations.
4.4 Measures are robust.	South Africa collects and reports measures in accordance with global strategies. MER indicators are primarily used to monitor progress; additional measures include KP size estimations, IBBS, and population-based surveys.
4.5 Information and processes are transparent and there are mechanisms for input and feedback from civil society, the private sector and donors.	SANAC commissions external reviewers to bolster transparency and credibility. Several perspectives are solicited during key informant interviews, an M&E Experts Workshop, a stakeholder validation workshop, document review, and epidemiological trend analysis.^6,10^

NGO, non-governmental organisation; KPs, Key populations; NSP, National Strategic Plan; FINCAP, Financial Capacity Building for Provinces; SANAC, South African National AIDS Council; PEPFAR, The U.S. President’s Emergency Plan for AIDS Relief; PrEP, Pre-exposure prophylaxis; DHIS, District Health Information System; NHLS CDW, National Health Laboratory Services Corporate Data Warehouse; PRC, Programme Review Committee; M&E, monitoring and evaluation; GAM, Global AIDS monitoring; SAG, South African government; MER, monitoring, evaluation, and reporting; IBBS, Integrated bio-behavioural surveys; WHO, World Health Organization; UNAIDS, The Joint United Nations Programme on HIV/AIDS; AFSA, AIDS Foundation of South Africa; NACOSA, Networking HIV and AIDS Community of Southern Africa; RMC, Resource Mobilisation Committee.

### Dimension 1: Political ownership and stewardship

The SAG has shown a high level of political ownership and stewardship. This political commitment is crucial as it underpins the subsequent three dimensions.

#### Criterion 1.1: Host government has a clear aspiration for what should be accomplished in each stage of programme development, implementation, and monitoring, generated with input from their own cities and rural areas, civil society, non-governmental organisations, and private sector, as well as their own citizens

Country ownership is demonstrated by the fact that South Africa has a clear aspiration for what should be accomplished, and strategies are informed by various stakeholders. The NSP has four strategic goals:

- Break down barriers to achieving outcomes for HIV, TB and STIs.
- Maximise equitable and equal access to services and solutions for HIV, TB and STIs.
- Fully resource and sustain an efficient NSP led by revitalised, inclusive and accountable institutions.
- Build resilient systems for HIV, TB and STIs that are integrated into systems for health, social protection, and pandemic response.^4^

In this regard, the NSP highlights the importance of KPs, as they are particularly affected. In this regard, South Africa is a signatory of a series of UNAIDS declarations, including the Fast-Track strategy to accelerate HIV epidemic control.^11,12^ These declarations and strategies from prominent HIV/AIDS multilateral organisations inform the creation of South Africa’s NSP, for which SANAC has been responsible since 2000. The current 2023–2028 NSP is aligned to the UNAIDS 2016 Political Declaration,^11^ aiming to significantly reduce new HIV infections, increase the number of people on antiretroviral therapy (ART), and decrease HIV-related deaths in the country, among other targets. Other policies or commitments made by South Africa to promote a tailored approach of the HIV response to the country’s most vulnerable populations include the National Sex Worker HIV, TB and STI Plan 2019–2022; the National LGBTI HIV Framework 2017–2022;^13^ and the 2013 Guidelines for the management of TB, HIV and STIs in Correctional Centres.^14^

As the fulcrum of the country’s response to the HIV epidemic, the NSP is generated with input from South African citizens, the private sector and development partners, the SAG, and civil society.^4^ The SANAC works closely with 18 organised sectors of society, including ‘PLHIV; lesbian, gay men, bisexual, transgender, and intersex people (LGBTI); youth; non-governmental organisations (NGOs); and people with disabilities’.^10^ Provincial consultations and national multi-stakeholder consultations were conducted to inform the current NSP (2023–2028).^4^ Additionally, SANAC requested public contributions both in person and through an online survey. The SANAC also coordinated various technical task teams to address key areas of the NSP. Ultimately, a team of technical experts comprised of the teams Prevention Technical Task Team, Social and Structural Drivers Technical Task Team, Human Rights Technical Task Team, and Strategic Information Technical Task Team synthesised the information garnered from various stakeholders. The draft of NSP was formally endorsed by Parliament and launched during World TB Day on 24 March 2023.^4^

#### Criterion 1.2: National plans are aligned to national priorities to achieve planned targets and results, with full costing estimates and plans incorporated

Country ownership is one of the focus areas of the new NSP.^4^ This includes advancing leadership and accountability as well as mobilising resources for the achievement of national targets.^4^ The inclusion of mid-term targets in the NSP provides additional opportunities for monitoring progress and making adjustments in service provision ahead of the end of the five-year NSP term.^4^ Additionally, the NSP includes full costing estimates not only to demonstrate financial transparency and guide sectoral and provincial government budgeting, but also to ensure that the NSP is fully resourced and sustained. Financial Capacity Building for Provinces^4^ has been introduced to strengthen country ownership by facilitating more accurate budgeting and tracking of expenditures at the provincial level:

Conditional grants are regulated through the Division of Revenue Act. Each grant has a Framework, which is an annexure in the Act Document. Funds are transferred to Provinces in line with Approved Business Plans. Provinces implement approved activities and report back to the transferring National Department. Challenges are usually experienced with compliance by provinces on provisions of the Act. Spending is usually not a major challenge due to high demand for financial resources or resources in general.^15^

#### Criterion 1.3: Host country (public and private sectors) is the architect that fully implements and provides oversight of national plan to achieve results and applies and scales up evidence-based best practices; this includes specific activities conducted by stakeholders in each stage from design to delivery of programmes

Another sign of a country-owned response is that both public and private stakeholders are intimately involved in the implementation of the NSP. Although support from international donors should not be minimised, South African organisations are primarily responsible for carrying out the NSP. The NSP outlines evidence-based strategies needed to accomplish targeted goals which include:

Scaling up HIV testing and Universal Test and Treat (UTT), investing in long-acting ART regimens and adherence support, voluntary medical male circumcision (VMMC), pre-exposure prophylaxis (PrEP), condom distribution, She Conquers and the Determined, Resilient, Empowered, AIDS-free, Mentored and Safe (DREAMS) programme for adolescent girls and young women (AGYW).^4^

### Dimension 2: Institutional and community ownership

In the words of internationally recognised HIV researchers and advocates, Chris Collins and Chris Beyrer: ‘country ownership must not come to mean simply government ownership; if it does, the voices of affected communities might not be heard, and accountability will suffer’.^16^ Civil society slogans, including ‘*Nothing for Us Without Us* and *Leave No One Behind’* reflect the ideal of amplifying marginalised voices. Involving communities in the response will strengthen the long-term sustainability of HIV services, especially among KPs.

#### Criterion 2.1: Host country institutions (inclusive of government, NGOs, civil society, and the private sector) constitute the primary vehicles through which health programmes are delivered and take responsibility for each programme

Communities have been the main responders in the HIV response through advocacy, community-led monitoring, and service delivery for over three decades. The active participation of communities in the HIV response is in line with commitment seven of the Global AIDS Monitoring (GAM) report. The GAM report showed that South Africa met the goal of having at least 30% of all service delivery be community-led by 2020.^10^ Some examples of South African organisations delivering HIV programmes are described below:

- AIDS Foundation of South Africa provided a core package of prevention programmes to reduce human rights-related barriers to HIV to over 25 000 AGYW in 2021. The organisation is overseeing the implementation of the sex work module in the current Global Fund grant.^6^
- Beyond Zero is present in seven provinces; in addition to providing services directly to MSM and transgender people, they also provided small grants and capacity building to 77 other South African organisations.^6^
- Networking HIV and AIDS Community of Southern Africa distributes its funds to 2500 community-based organisations (CBOs) in seven provinces to deliver services to sex workers. In 2021, NACOSA’s services reached 40 352 sex workers, 40 618 children and youth, 65 301 AGYW, and 9979 PWUD. The organisation also provides economic empowerment and other structural services to improve the health, psychosocial, and socio-economic well-being of KPs.^6^

These three organisations, as well as others, are supported through conditional grants from PEPFAR and the Global Fund, not from SAG funds.^6,10^ The reliance on donor funding for the implementation of these crucial activities is of concern, given the uncertainty of future support. Ideally, the SAG should have adequate resources to demonstrate ownership. However, due to competing health priorities in a country with a quadruple burden of disease,^17^ donor support may be inevitable. The National Health Insurance System that is being pioneered in South Africa has the potential to mitigate the situation.^18^ Moving towards a more self-sufficient and localised funding mechanism that is integrated into the national systems would ensure the sustainability of community-based efforts and, ultimately, of the HIV response.

Community leaders of KP groups and PLHIV also contribute substantially to the national response through community-led monitoring of HIV services and holding the SAG accountable for the provision of high-quality services. For example, Ritshidze is a community-led monitoring system established by organisations representing PLHIV. Through this mechanism – which is utilised in 400 clinics and community healthcare centres across 27 districts in eight provinces – the community can systematically identify and document HIV service delivery and monitor challenges.^6^ Additionally, the establishment of the High Transmission Area programme and the use of differentiated service delivery models to address the gaps in ART signal an increased focus on KPs. However, programme data systems need to be reviewed and strengthened to elicit quality KP data.^6,18^

#### Criterion 2.2: Host country institutions adopt and implement transparent, evidence-based policies/regulations for priority areas that align with national plans

The country has policies and legislation that support community service delivery approaches spearheaded by NGOs.^4^ A partnership between the Department of Correctional Services and NGOs – including Aurum, TB/HIV Care, and Right to Care – aims to improve ART delivery among people who are incarcerated.^6,10^ Additionally, TB/HIV Care and the South African Network of People who Use Drugs successfully developed training materials on the rights of PWUD and the country’s first harm reduction policy became a pillar of the National Drug Control Plan.^6^ Further, the South African Policy Framework and Strategy for Ward Based Primary Health Care (PHC) Outreach Teams has been put in place to provide the directive for the improvement of community health workers.^19^ The coverage of services provided for KPs by the NGOs alluded to in this manuscript is evidence of real-world impact on populations that would have otherwise not received the services. However, more needs to be done as great policies have not necessarily been translated into great implementation.^10^

Capacity strengthening programmes on human rights, non-discrimination, violence prevention, and gender-based violence are provided to law enforcement personnel, HCWs, and judiciary members at both the national and subnational levels.^3^ The country has embarked on service quality assessments and facility accreditations to address discriminatory laws. South Africa has implemented policies addressing punitive legal and policy environments for fostering a safe environment for PLHIV, although only 54% of PLHIV had knowledge of the laws that protect them from discrimination and only 56% of PLHIV were aware of organisations that can protect their rights.^20^ However, stigma, discrimination and criminalisation persist among KPs,^20,21,22^ despite availability of policies. This demonstrates the need for having feasible plans for translating policy into action.^4^

#### Criterion 2.3: Host country institutions manage funds

The SAG largely relies on donor funds for KP programming led by CBOs. For example, Beyond Zero and NACOSA serve as grant managers, determining which sub-recipient organisations will receive funding towards KPs programmes across South Africa.^10^ Although the current funding trends demonstrate a continued commitment of the SAG to be the primary funder of HIV programmes, the government’s funding prioritises treatment over prevention, as well as the general population over KPs. To further empower local institutions to manage funds, PEPFAR South Africa increased funding to local CBOs from 73% in 2018 to 80% in 2019.^6^ Although support from international donors is commendable, the SAG should ensure adequate domestic resources to ensure country ownership and sustainable programming.

### Dimension 3: Capabilities

#### Criterion 3.1: Host country has effective workforce, organisations and systems at all levels able to perform activities and carry out responsibilities that achieve priority health outcomes

The World Health Organization (WHO) African Region has historically faced chronic healthcare worker (HCW) shortages for the provision of routine health and HIV services. The SAG and international donors have continued to invest in human, financial, and organisational resources. The funding is aimed at empowering local CBOs to deliver HIV services, which in the long run will contribute to sustainability. South Africa is one of three African countries with a favourable HCW-to-population ratio of 60.46 skilled health professionals per 10 000 population in 2016.^6^ However, increasing the number of PHC nurses skilled to administer ART, address STIs, and uphold infection control standards is a focus of the NSP.^4^ To accomplish this, South Africa has a Human Resources for Health (HRH) programme aimed at skilling the health workforce for increased efficient and effective HIV service delivery.^23^ One output of the HRH programme is 23 000 nurses trained in nurse-initiated management of ART (NIMART).^23^ Additionally, the SAG is working with PEPFAR South Africa to ‘recruit highly-skilled, short-term Peace Corps volunteers to support in capacity building, change management, monitoring and evaluation (M&E), programmatic support, and other high-impact areas’.^6^ Community health workers (CHWs) are also crucial members of the healthcare workforce. In addition to investing in their training and integration into the health system, recent evidence also supports improved geographical allocation to areas most in need.^4,10^ Lastly, the effects of the COVID-19 pandemic on the South African healthcare system – including the availability of skilled health professionals to provide HIV services amidst task shifting and competing priorities – should not be understated.^10^

South Africa has also experienced procurement and supply issues. For example, there have been challenges related to PrEP affordability and accessibility due to high out-of-pocket costs, poor awareness, stigma, discrimination, as well as stockouts requiring more commitment of local resources.^24,25^

#### Criterion 3.2: National coordinating bodies and local institutions having ability to gather and analyse epidemiological and programme data to plan and measure programme progress and results

South Africa is committed to building robust information management and monitoring systems. A range of HIV surveillance systems provide the strategic information necessary to guide the HIV response.^4^ For example, the District Health Management system (DHIS) provides facility registers and treatment cascade data (e.g., viral load testing; CD4 count results; ART initiation, retention, and adherence). Within the DHIS, Three Interlinked Electronic Registers.Net (TIER.net) and the Patient Monitoring System capture patient-level data at entry into care and longitudinally using electronic medical records.^4,10^ The National Health Laboratory Services (NHLS) has their own Corporate Data Warehouse which houses laboratory information, financial information, and national statistics. Despite not being otherwise linked, the GAM report harmonises and triangulates these different data sources to provide evidence of progress towards the country’s Fast-Track commitments.^6,10^

Numerous gaps to strengthen and harmonise data systems exist. For example, Synch and other important data sets that measure programme progress do not interface with existing data sets and are not linked and until recently have not been country-owned and have been set up in parallel. Further, the National Department of Health (NDoH) still does not collect data disaggregated by KPs due to the lack of unique identifiers. Substantial evidence supports the move toward unique identifiers to preserve privacy while allowing for longitudinal case-based surveillance. It should also be noted that the data that are being collected have several data quality limitations, such as completeness, timeliness, integrity and reliability. Due to staff shortages, sometimes the data are not captured on time from the paper-based registers into the electronic system. Late data capturing affects reporting data on time to be used to inform planning and programming.^6,10^

#### Criterion 3.3: Host country institutions have the capabilities required to perform or oversee activities for programmes

The previous criteria have demonstrated the ability of South African institutions to perform and oversee programmatic activities. Furthermore, the Programme Review Committee brings together programme implementers in South Africa to address technological advances during NSP implementation. Regarding oversight, South Africa has robust M&E strategies and plans, and dedicated personnel at SANAC to carry out this out. National and provincial M&E personnel are responsible for producing quarterly factsheets, which highlight progress towards quarterly targets. SANAC is also responsible for coordinating the compilation of the GAM report which documents the country’s progress.^6,10^ Although the roles are clearly delineated; there is a need to strengthen accountability mechanisms for reporting.

#### Criterion 3.4: Host country institutions have the ability to dynamically modify programmes based on evidence and feedback from monitoring processes

In addition to M&E plans, SANAC is developing the Situation Room – an interactive software platform that will enable global health actors to use the HIV-related data from various stakeholders for real-time decision-making. The platform will enable visualisation of the national and subnational health data and show progress towards NSP targets. The web-based KP Master Tool, developed by SANAC in 2017, will feed information into the Situation Room.^26^ One of the primary challenges is the lack of data access and integration in the Situation Room, given how expensive (licensed per user) the software used for data analysis and visualisationis.^27^

### Dimension 4: Mutual accountability, including finance

#### Criterion 4.1: Host country is responsible to country citizens and international stakeholders for achieving planned results

In addition to SANAC’s role of fostering dialogue between various stakeholders, the Global Fund Country Coordinating Mechanism (CCM) also plays an oversight role. The CCM meets quarterly to review progress made by principal recipients and provides guidance where there are bottlenecks in implementation.^4,10^ Further, a quarterly Operational Performance and Efficiency Coordination meeting is conducted to promote mutual accountability between Global Fund recipients. Operation Phutuma conducts meetings with a multi-disciplinary team of HCWs to deliberate on the performance of indicators, and to identify challenges and improvements.^28^

#### Criterion 4.2: Host government is responsible for financing and financial stewardship over health

HIV consistently receives the most international aid of any single disease (with the exception of COVID-19 emergency funding in 2020), amounting to $9 billion in 2020 alone.^29^ Substantial resources have been allocated to combat the HIV epidemic in South Africa, yet incidence remains unacceptably high. The 30% steady decline in HIV incidence is not sufficient.^6,10^ South Africa still has more than double the number of new infections per annum (about 200 000) compared to the target of 100 000 new infections per annum.^10^ In the fiscal year 2019–2020, approximately R37.6 billion ($2.5 million) was spent to address HIV.^5,29^ However, nearly 75% of the 2019–2020 HIV expenditure went towards treatment programmes, leaving only a quarter of the funding for prevention (8%), testing and counselling (5%), social protection and economic support (6%) programmes, and systems strengthening (7%) combined.^29^ Overall, the SAG is the largest spender (69%), funding the HIV response using domestic public revenue generated by the National Treasury.^6,29^ SANAC is responsible for mobilising these resources, both domestically and internationally. As such, the SAG obtains additional funding to supplement government resources and pilot innovative interventions from external development partners.^30^ The contributions made to the 2019/2020 expenditure included: 24% from PEPFAR, 6% from the Global Fund and other international donors and 1% from the private sector.^29^

The Resource Mobilisation Committee (RMC) provides comprehensive country-wide oversight of all funding arrangements related to NSP implementation. The RMC assesses funding opportunities, aligns with NSP goals and objectives, recommends NSP funding, and identifies interventions to assist in the allocation of sufficient funding. Unfortunately, the 2020 National Spending Assessment revealed that KPs receive minimal funding from the HIV response.^6^ However, despite the constrained fiscal environment in recent years, the SAG has continued to demonstrate financial commitment to HIV programmes, with funding for HIV growing to be more than the overall health budget over the last nine years.

#### Criterion 4.3: Explicit roles and responsibilities are described with appropriate management of performance in place

There appears to be country ownership with respect to clear-cut roles and responsibilities in the HIV response. Among others, SANAC advocates for proposals from civil society, provincial and local governments, the private sector, technical experts, and partners regarding policy or programmatic changes. Further, SANAC engages all organisations, partners, and stakeholders in the national response to ensure implementation of the multisectoral approach, co-ownership, and accountability.^3^ In addition, SANAC works with various government departments such as health, education, social development, justice, and correctional services to ensure successful implementation of the NSP.^6^ Furthermore, SANAC works closely with international donors as well as multilateral organisations such as UNAIDS, WHO, and the United Nations Population Fund. The collaboration and cooperation between government, civil society, development partners, private sector, donors and multilateral organisations allows for an optimal allocation of responsibilities as well as expansion of the national response.

#### Criterion 4.4: Measures are robust

There is also a national framework and scorecard that specifies processes, data sources, human resources, stakeholders, and related items for the NSP. Strategic information serves to assess the country’s performance toward set targets.^3^ The measures used to evaluate the NSP are in alignment with monitoring, evaluation, and reporting (MER) indicators and other externally validated, robust measures used to compare metrics over time and space. Examples of data collected include KP size estimations, integrated bio-behavioural surveys (IBBS), and population-based surveys for tracking and managing the epidemic. Additionally, the country utilises data from the Global Fund performance framework to track performance at outcome and impact level indicators and guide modelling projections (e.g., Thembisa Model). The indicators contained in the NSP are reported annually to UNAIDS resulting in the GAM report published annually on 01 December.

#### Criterion 4.5: Information and processes are transparent and there are mechanisms for input and feedback from civil society, the private sector and donors

To ensure mutual accountability, and transparency, there are structures that collectively monitor progress and implementation of the NSP. The SANAC commissions external evaluators to conduct the NSP Mid-Term Review and End-Term Review.^15,31^ The reviews take a highly consultative and participatory approach in which several stakeholders contribute, with a central multisectoral steering committee providing oversight and guidance. Before finalisation and publishing of reports, validation meetings/workshops are held where stakeholders give direct feedback on the findings of the report as well as provide information that might be lacking to be included in the reviews.

## Discussion

In this article, we analysed South Africa’s ownership of the national HIV response using published and unpublished reports and literature, as well as the authors’ subject matter expertise. The analysis revealed South Africa’s substantial progress towards achieving country ownership of the national HIV response. The recommendations that follow include actionable steps that would allow South Africa to progress further along the spectrum of country ownership.

### Dimension 1: Political ownership and stewardship

Political commitment and stewardship are fundamental to the HIV response.^4^ In this regard, South Africa has demonstrated notable commitment to political ownership and stewardship. However, gaps still exist in policy implementation and local accountability mechanisms.^10^ Effective implementation of policies, institutional and legal frameworks in the communities where PLHIV access services can be addressed through making funds available at CBOs who work directly with PLHIV.^10^ South Africa also needs to intensify the uptake of evidence-based innovative interventions targeted at addressing context-specific issues.

For South Africa to fully own its HIV response and achieve the largest population-level impact, a better understanding of the HIV burden and patterns of HIV transmission among KPs is required. Not addressing the unmet HIV prevention and treatments needs of KPs has negative epidemic consequences.^10,31^ For example, it is estimated that nearly 50% of HIV infections between 2020 and 2029 would stem from the unmet HIV prevention and treatment needs of FSWs and their clients.^31^

### Dimension 2: Institutional and community ownership

The limited funding for KP programmes is a critical issue; more resources for KPs need to be lobbied to contain HIV. These resources should prioritise integrated health service delivery and differentiated models of care. South Africa can also utilise organisations trusted by KPs to promote a differentiated approach to service delivery and improve engagements as one of the ways to adopt and implement transparent, evidence-based policies/regulations for priority areas that align with national plans.^10^ Further, modelling studies assessing the programme impacts of prioritising KPs need to be conducted. For example, the impact of PrEP on reduction in HIV incidence among KPs should be determined.^24,25^ A multisectoral approach is critical more than ever before ahead of the 2030 global goal to end HIV as a public health threat. To advance the cause of protecting KPs, the Department of Social Development could assist in addressing the social and economic drivers of HIV (e.g., gender-based violence, lack of education, poverty, stigma, discrimination, unemployment, etc.). Additionally, the Department of Justice should focus on law reform investments and working with communities to change social norms.^10^

### Dimension 3: Capabilities

The review highlighted a need for capacitation and sensitisation of HCWs for South Africa to adequately respond to the HIV.^4,10^ While short-term Peace Corps volunteers maybe helpful in combating the South African HIV epidemic, they are not the solution to progressing along the continuum of country ownership. It is encouraging to see buy-in from the SAG in this initiative, however, and the goals of this partnership are supportive of Dimension 3. One of the central messages of this review is that the capacitation of local CBOs will go a long way in ensuring a sustainable response. Some of the recommendations to improve on Dimension 3 should include:

Provision of additional human resources and skills support for the development of activity-based costing of M&E plans; ensuring adequate availability of human resources to support the supply chain and procurement system and consider a decentralised procurement system and integration with relevant programmes and departments.^4.^

South Africa needs to prioritise building robust information management and monitoring systems. There are several considerations that may enable national coordinating bodies and local institutions to gather and analyse epidemiological and programme data to plan and measure progress. Considerations include understanding how existing data systems can be linked or consolidated, improving data quality and assurance, and establishing both data repository and surveillance to provide the strategic information necessary to guide the HIV response.^4^

A centralised system to gather and monitor KP data would allow South African institutions to respond to the distinct needs of these populations more adequately. The DHIS should have HIV-specific unique identifier codes (UIC) for KPs to link their records for testing, treatment, and laboratory services across geographies and programmes. Unique identifier codes are already utilised for all individuals to protect privacy, but at present these UICs do not identify KPs or other marginalised identities.^32^ South Africa may need to learn from countries that have implemented KP UIC in routine data systems, such as Ghana.^33^

### Dimension 4: Mutual accountability, including finance

In the absence of a massive decline in the HIV incidence rate, the absolute numbers of people acquiring HIV will remain alarmingly high. Providing decades-long treatment to ever-growing numbers of PLHIV will remain a financial responsibility for the government, with overall consequences on the ability to achieve epidemic control.^32^ Mutual accountability and multisectoral response are encouraged, as no single department or sector can achieve epidemic control alone.^4^ The point is to hold all stakeholders accountable rather than expecting the SAG to sustain the response all by themselves. Buy-in from the private sector and local businesses to participate in financing the HIV response is required to achieve mutual collaboration between government and non-government organisations. Funding that is given to empower local CBOs will contribute to sustainability. Previously, international funding was given to international organisations to deliver services in South Africa which affected sustainability. Although the SAG continues to make strides in investing in human, financial, and organisational resources it does not have adequate resources due to competing health priorities. HIV prevention activities suffer most from constrained SAG funding, as do populations that are at higher risk of HIV acquisition. As such, there is a need to develop a plan for transitioning KP and prevention programming from primarily donor-funded to government funded. South Africa should also consider the utilisation of the National Health Insurance to expand the financing for the HIV response. In light of the declining donor HIV funding, a strategic plan to combat this can be developed in consultation with all relevant stakeholders. To ensure that information and processes are transparent, annual National Spending Assessment reports need to be institutionalised.

### Strengths and limitations

The findings of this review should be interpreted in the context of the strengths and limitations of the nature of the review. The authors had many consultative interviews with stakeholders (some of whom are co-authors) throughout the compilation of this manuscript. The manuscript also benefited from the inputs of the global community as it was presented at AIDS 22 in Montreal, Canada at the symposium entitled ‘Quest to Reach HIV Epidemic Control: Country Ownership and Sustainable Programming in South Africa’. The conceptual framework used to evaluate country ownership was developed in 2012 by the GHI. Recognising the progress made in country and global responses to HIV, the framework could be improved and updated. The criteria for each dimension often overlapped, making it difficult for the authors to determine the appropriate location for evidence and discussion. Further, although Dimension 2 considers CBOs and civil society involvement, the framework has a strong focus on the government’s role. Another limitation of this review is the nature of the framework’s subjective inputs; although the inter-disciplinary author team engaged in many discussions about each of the dimensions, our points of view are influenced by our experience and knowledge, and therefore are up for debate. Having more specific criteria or benchmarks for objectively deciding when a given aspect of country ownership has been achieved would have improved the objectivity of our review. Our manuscript sought to give a high-level view of the status quo, but we encourage follow-up manuscripts to delve deeper into certain areas with subject matter experts.

## Conclusion

While country ownership is critical for South Africa, it is also important to highlight some of the unique challenges the country faces. Sustainability remains a critical issue for South Africa due to reliance on donor funds. Additionally, the availability of donor funding may disincentivise South Africa to accept sole financial responsibility for its HIV response. However, continued donor support and investments in technical capacity may be necessary to avoid a rapid transition into full country ownership.^4^ This is also supported in the current NSP 2023–2028 as the ‘People’s NSP’.^4^ We also believe that the management of the transition from reduction of donor support should be carefully managed. Trying to progress too rapidly – by not allowing any international donor support, for example – would likely prove disastrous. The principle of gradual divestment is also reflected by Gavi, The Vaccine Alliance’s approach to providing countries with financial and technical support for vaccines until they have fully ‘graduated’. We are suggesting a similar model for HIV resources, so that programmes are not disrupted during the funding transition. Additionally, it may not be realistic to rapidly transition all international donor-supported programmes and services to South Africa for reasons mentioned above. Rather, capacitation of local CBOs and gradual ownership is preferred to optimise sustainability. South Africa should also maximise on strategic information to strengthen reporting, guide decision-making, and improve inefficiencies. Other barriers to realising country ownership include unfavourable economic situations such as poverty and unemployment, political will and commitment, prioritisation of the response by the government and its in-county stakeholders and competing government priorities.

This article draws on the most recent evidence regarding the South African HIV epidemic and the extent to which the response is country-owned. We provided practical, evidence-based recommendations on how South Africa can progress further along the spectrum of country ownership. Encouraging continued local investment in financial, human, and organisational resources should be a priority for South Africa.
